# Discrimination in Healthcare Settings is Associated with Disability in Older Adults: Health and Retirement Study, 2008–2012

**DOI:** 10.1007/s11606-015-3233-6

**Published:** 2015-03-13

**Authors:** Stephanie E. Rogers, Angela D. Thrasher, Yinghui Miao, W. John Boscardin, Alexander K. Smith

**Affiliations:** 1 Department of Medicine, Division of Geriatrics, University of California, San Francisco, CA USA; 2 Veterans Affairs Medical Center, San Francisco, CA USA; 3 Department of Health Behavior, University of North Carolina Gillings School of Global Public Health, Chapel Hill, NC USA

**Keywords:** disability, discrimination, geriatrics

## Abstract

**BACKGROUND:**

As our society ages, improving medical care for an older population will be crucial. Discrimination in healthcare may contribute to substandard experiences with the healthcare system, increasing the burden of poor health in older adults. Few studies have focused on the presence of healthcare discrimination and its effects on older adults.

**OBJECTIVE:**

We aimed to examine the relationship between healthcare discrimination and new or worsened disability.

**DESIGN:**

This was a longitudinal analysis of data from the nationally representative Health and Retirement Study administered in 2008 with follow-up through 2012.

**PARTICIPANTS:**

Six thousand and seventeen adults over the age of 50 years (mean age 67 years, 56.3 % female, 83.1 % white) were included in this study.

**MAIN MEASURES:**

Healthcare discrimination assessed by a 2008 report of receiving poorer service or treatment than other people by doctors or hospitals (never, less than a year=infrequent; more than once a year=frequent). Outcome was self-report of new or worsened disability by 2012 (difficulty or dependence in any of six activities of daily living). We used a Cox proportional hazards model adjusting for age, race/ethnicity, gender, net worth, education, depression, high blood pressure, diabetes, cancer, lung disease, heart disease, stroke, and healthcare utilization in the past 2 years.

**KEY RESULTS:**

In all, 12.6 % experienced discrimination infrequently and 5.9 % frequently. Almost one-third of participants (29 %) reporting frequent healthcare discrimination developed new or worsened disability over 4 years, compared to 16.8 % of those who infrequently and 14.7 % of those who never experienced healthcare discrimination (*p* < 0.001). In multivariate analyses, compared to no discrimination, frequent healthcare discrimination was associated with new or worsened disability over 4 years (aHR = 1.63, 95 % CI 1.16–2.27).

**CONCLUSIONS:**

One out of five adults over the age of 50 years experiences discrimination in healthcare settings. One in 17 experience frequent healthcare discrimination, and this is associated with new or worsened disability by 4 years. Future research should focus on the mechanisms by which healthcare discrimination influences disability in older adults to promote better health outcomes for an aging population.

**Electronic supplementary material:**

The online version of this article (doi:10.1007/s11606-015-3233-6) contains supplementary material, which is available to authorized users.

## INTRODUCTION

With the changing landscape of an aging American population, healthcare institutions are increasingly challenged to improve the health and dignity of older adults. The Institute for Healthcare Improvement recently began its Triple Aim quality improvement initiative focusing on simultaneously improving the patient experience, improving health, and cutting costs^1^; discrimination in healthcare settings has the potential to impact all three components of the Triple Aim. First, patients may accurately perceive that they receive worse treatment, leading directly to worse health outcomes. Second, patient experiences of discrimination in healthcare settings may decrease patient satisfaction, contributing to avoidance of beneficial medical care or treatment noncompliance.^2^–^4^ Third, while some may question the responsibility of physicians and health systems to address social determinants of health, eliminating discrimination in healthcare settings is incontrovertibly their responsibility.^5^ Finally, health systems will have a strong economic incentive to reduce the perception of discrimination in healthcare settings, as Medicare payments under the Affordable Care Act will be tied to patient satisfaction and quality of care delivered to older patients.

To our knowledge, no studies have examined the health effects of discrimination specifically in healthcare settings on older adults. Studies in the general population have shown an association between self-reported everyday discrimination and adverse health outcomes, including increased rates of depression and mental health disorders, high blood pressure, and cardiovascular outcomes,^6^–^8^ while a relatively smaller number have explored these relationships in samples of older adults.^9^, ^10^ Older adults who experience everyday discrimination are more likely than those who do not report such experiences to have poorer mental health^11^–^13^ and physical health, including mortality,^14^–^17^ as well as the use of fewer preventive health services.^18^–^20^ These findings suggest that discrimination may be an important social determinant of older adult health.

The purpose of our study was to assess the prevalence of healthcare discrimination using a nationally representative sample of adults aged 50 years and older, and examine its relationship with new or worsened disability. Disability is a common end-result of acute and chronic conditions in the elderly. Because the effects of discrimination in healthcare settings may cut across multiple health conditions, we hypothesized that discrimination in healthcare settings may result in early functional decline in older adults.

## METHODS

### Participants

We used data from the 2008, 2010, and 2012 waves of the Health and Retirement Study (HRS). HRS is a nationally representative longitudinal studyof American adults over the age of 50 that is conducted every 2 years and assesses important aspects of the aging process.^21^ In addition to the primary survey administered to 15,649 adults in our study sample, HRS selects a random sample of participants to complete supplementary surveys on additional topics. The auxiliary 2008 HRS Psychosocial Leave-Behind Participant Lifestyle self-administered mail-in survey was assigned to 7,018 participants, after excluding 8,101 adults who were randomly assigned to a different mail-in survey and 530 participants who were not eligible.^22^ Of these, 764 (11.5 %) participants did not return or complete the questionnaire. Of the 6,254 remaining participants, 108 (1.4 %) were excluded because they did not respond to the question regarding healthcare discrimination. The 108 participants who were excluded for not answering the study question were older than those who responded (mean 71.8 vs. 66.9 years, *p* = 0.05), and had a lower proportion of racial/ethnic minority than white participants (67.7 % vs. 83.1 %, *P* < 0.001). Of the remaining 6,146 participants, 129 participants were excluded from the final analysis because we did not have follow-up information for them in 2012. Our final sample was 6,017 (96.2 % of eligible participants).

### MEASURES

#### Independent Variable

The healthcare discrimination item was taken from the Everyday Discrimination Scale (EDS), a well-established measure that has demonstrated good reliability in samples of older adults.^16^, ^23^ The item of interest asked, “In your day-to-day life, how often have any of the following things happened to you? You receive poorer service or treatment than other people from doctors or hospitals.” Other questions in the EDS asked: Are you are treated with less courtesy or respect? Do you receive poorer service at restaurants or stores? Do people think you are not smart? Do people act afraid of you? And, have you been threatened or harassed? We decided to use the single item because of our specific interest in experiences of discrimination with the healthcare system, not general experiences of discrimination. Response categories were never, less than once a year, a few times a year, a few times a month, at least once a week, or almost every day. We classified the responses into three groups: never experienced health care discrimination; infrequently experienced (less than once a year); and frequently experienced (a few times a year to almost every day), as previous research suggests that increasing frequency of discrimination can lead to worse health outcomes.^24^ If they responded affirmatively to any item on the Everyday Discrimination Scale, participants were asked to mark one or more reasons why they believed the discrimination happened to them (e.g., age, gender, race, physical disability).

#### Primary Outcome

The outcome was report of new or worsened disability at 4 years. To determine Activities of Daily Living (ADL) functioning, participants were asked if they had difficulty in any of six activities: ambulating, bathing, dressing, eating, toileting, and transferring. Participants who responded that they had difficulty were asked if they required assistance for the ADL. New or worsened disability was defined by a progression from no ADL difficulty or dependence in 2008 to ADL difficulty or dependence in 2010 or 2012 (new disability), or ADL difficulty in 2008 to ADL dependence in 2010 or 2012 (worsened disability).

#### Covariates

Age, race/ethnicity, and educational attainment were obtained by self-report. The HRS collapses patients of American Indian, Alaskan Native, Asian, and Pacific Islander race into “other race” to protect participant confidentiality. Net worth was determined by asking participants to report their debts and assets. Comorbid conditions, including hypertension, diabetes, cancer, chronic lung disease, heart conditions, and stroke, were evaluated by asking the participants if a physician had ever told them that they had the condition. These comorbidity questions are strongly associated with disability in late life.^25^ Cognitive impairment was determined using the Telephone Interview for Cognitive Status (TICS).^26^ Depression was determined by positive responses to three or more symptomatic items on the eight-item Center for Epidemiological Studies Depression Scale.^27^, ^28^ Participants reported if they had ever smoked or were currently drinking any quantity of alcohol. Body mass index was determined using the participants’ subjective reports of current weight and height. Those participants who rated their vision and hearing as fair to poor were reported as having a visual or hearing impairment. Healthcare utilization was determined by asking the patients to recall how many times in the past 2 years they had been hospitalized (never/1/2+), if they had ever been a patient overnight in a nursing home (yes/no), if they had outpatient surgery (yes/no), and how many clinic or emergency department visits they had (never/<20/>20) and if they used home health services (yes/no). Use of preventive health services during the previous 2 years (flu shot, cholesterol testing, and mammography) was assessed by patient report. Mortality during the study period was determined from reports from next-of-kin and linkage to the National Death Index.

### Statistical Analysis

*T*-tests and chi squares were used to characterize the samples reporting never, infrequently, and frequently experiencing healthcare discrimination. Reported reasons for experiencing healthcare discrimination are described. We report the top reasons for experiencing healthcare discrimination among selected subgroups (age group, gender, race/ethnic groups). We then used Kaplan-Meier plots to examine the relationship between the report of experiencing healthcare discrimination and disability over time. To determine the independent contribution of reported healthcare discrimination to the onset of new or worsened disability over time, we used Cox Proportional Hazards models, censoring patients that died during the study and those alive at the end of the study. Multivariable Cox models were adjusted first for age alone, then for sociodemographic variables (age, gender, race/ethnicity, net worth, educational attainment), and comorbidities (high blood pressure, diabetes, cancer, lung disease, heart disease, stroke, and depression). Because there was concern that more exposure to the healthcare system increased the likelihood for perceiving discrimination, the final models also adjusted for all measures of healthcare system utilization in the past 2 years. In a sub-analysis, we analyzed the association between the entire EDS and EDS measures other than healthcare discrimination and the development of disability over time. First, we compared the development of disability among participants that reported frequent discrimination in any setting (any response of “a few times a year” or more to any of the six EDS items) to those who never experienced discrimination. Secondly, we compared the development of disability among participants that reported frequent discrimination outside of healthcare settings (any response of “a few times a year” or more frequently to any of the five EDS items excluding the healthcare item) to those who never experienced discrimination. All analyses used survey weights provided by HRS to account for the complex survey design and unequal probability of participant selection. Analyses were performed using STATA 12 (College Station TX).

## RESULTS

### Sample Characteristics

Characteristics of the study participants are presented in Table 1. The mean age was 67 years, 83.1 % were white and 56.3 % were women. In this group, 18.6 % experienced healthcare discrimination. Of these, 12.6 % experienced healthcare discrimination infrequently, while 5.9 % experienced discrimination frequently. Compared to the participants who did not experience healthcare discrimination, those who reported experiencing it frequently were younger, non-white, were less likely to be married or partnered, had lower net worth, were more likely to have less than a high school education, were more depressed, diabetic, had more chronic lung disease, were more likely to be a current smoker, had a higher BMI, had vision impairment, had more ADL difficulty and dependence at baseline, and had increased utilization of the healthcare system (Table 1).

**Table 1.** Characteristics of Participants

Characteristics	Overall (*N* = 6017)	Never experienced discrimination (*n* = 4958)	Infrequently experienced discrimination (*n* = 699)	Frequently experienced discrimination (*n* = 360)	*p* value
Age, yr					
Mean (SD)	67.0 (9.7)	67.4 (9.7)	65.2 (9.5)	65.4 (9.1)	<0.001
50–60 years	32.5 %	30.4 %	42.1 %	40.4 %	<0.001
61–70 years	34.1 %	34.6 %	30.7 %	34.2 %	
>70 years	33.4 %	35.0 %	27.2 %	25.5 %	
Female	56.3 %	57.2 %	51.2 %	53.9 %	0.06
Ethnicity					<0.001
White	83.1 %	84.2 %	81.6 %	71.5 %	
African American	8.5 %	7.6 %	10.6 %	15.9 %	
Latino	6.8 %	6.6 %	6.3 %	9.6 %	
Other	1.7 %	1.6 %	1.6 %	3.0 %	
Immigrant	7.8 %	8.0 %	6.0 %	8.8 %	0.30
Married or partnered	64.1 %	64.8 %	64.7 %	53.8 %	0.002
SES measures					
Less than high school education	20.5 %	20.4 %	13.7 %	35.8 %	<0.001
Net worth					
Mean (SD)	509,982	534,135	466,308	271,297	<0.001
Median (1st, 3rd quartile)	(1,123,810) 217,540 (55,000, 585,000)	(1,169,770) 239,500 (64,000, 607,000)	(936,110) 181,000 (42,239, 560,000)	(757,835) 80,000 (8,320, 289,316)	
Comorbidities					
Hypertension	56.4 %	55.9 %	56.1 %	65.2 %	0.03
Diabetes	19.1 %	18.2 %	19.7 %	30.7 %	<0.001
Cancer	14.8 %	14.7 %	15.1 %	15.9 %	0.83
Chronic lung disease	10.9 %	10.3 %	10.9 %	18.4 %	0.001
Heart condition	24.3 %	23.4 %	27.9 %	29.0 %	0.03
Stroke	7.9 %	7.8 %	7.0 %	11.7 %	0.04
Cognitive impairment	2.6 %	2.5 %	2.5 %	3.7 %	0.42
Depression	20.3 %	17.6 %	24.8 %	48.7 %	<0.001
Other health conditions					
Ever smoked	57.6 %	56.8 %	59.0 %	64.8 %	0.04
Current smoker	14.1 %	13.6 %	13.1 %	22.6 %	<0.001
Alcohol use	54.7 %	55.0 %	58.0 %	43.5 %	<0.001
BMI					
Mean (SD)	28.3 (5.9)	28.1 (5.6)	28.7 (6.5)	30.2 (7.4)	<0.001
Median (1st, 3rd quartile)	27.4 (24.3, 31.3)	27.4 (24.2, 31.1)	27.8 (24.4, 31.9)	28.7 (25.1, 33.1)	
Vigorous activity					0.12
Every day	3.5 %	3.4 %	3.2 %	4.3 %	
>1 per week	22.0 %	22.1 %	25.2 %	13.9 %	
1 per week	9.5 %	9.6 %	10.1 %	8.1 %	
1–3 per month	7.1 %	7.2 %	6.8 %	6.3 %	
Never	57.9 %	57.7 %	54.8 %	67.5 %	
Hearing impaired	20.0 %	19.4 %	22.3 %	24.8 %	0.04
Vision impaired	19.6 %	18.3 %	21.4 %	33.7 %	<0.001
ADL difficulty	15.4 %	14.0 %	16.3 %	33.0 %	<0.001
ADL dependence	5.5 %	4.9 %	6.1 %	11.2 %	0.001
Measures of contact with the health care system					
Number of times seen a physician in last 2 years					
Mean (SD)	10.2 (18.6)	10.0 (19.0)	10.0 (14.4)	14.0 (21.2)	0.02
Median (1st, 3rd quartile)	6 (3, 10)	6 (3, 10)	6 (3, 12)	8 (4, 16)	
Never	4.3 %	4.2 %	4.1 %	6.0 %	0.001
1–20 times	84.1 %	85.0 %	82.5 %	74.8 %	
>20 times	11.7 %	10.9 %	13.4 %	19.3 %	
Been hospitalized in last 2 years	25.4 %	25.1 %	22.7 %	34.1 %	0.002
Been hospitalized in last 2 years					
Never	74.7 %	74.9 %	77.3 %	66.1 %	<0.001
Once	15.4 %	15.4 %	14.8 %	16.3 %	
≥2 times	9.9 %	9.7 %	7.9 %	17.6 %	
Ever overnight in nursing home in last 2 years	1.9 %	1.9 %	2.0 %	2.2 %	0.92
Outpatient surgery in last 2 years	22.6 %	22.3 %	23.0 %	24.8 %	0.67
Use of home health services	6.8 %	6.5 %	5.5 %	14.4 %	<0.001
Preventive service in the past 2 years					
Flu shot	64.1 %	64.7 %	63.5 %	56.8 %	0.04
Cholesterol testing	84.9 %	85.2 %	85.4 %	80.1 %	0.22
Mammography*	71.1 %	71.8 %	71.0 %	64.0 %	0.79

Reported values incorporate survey weights to account for the complex survey design

* Mammography use was assessed in women who did not have breast cancer and were willing to complete supplementary module (*n* = 303)

### Reasons for Experiencing Healthcare Discrimination

**Figure 1.**
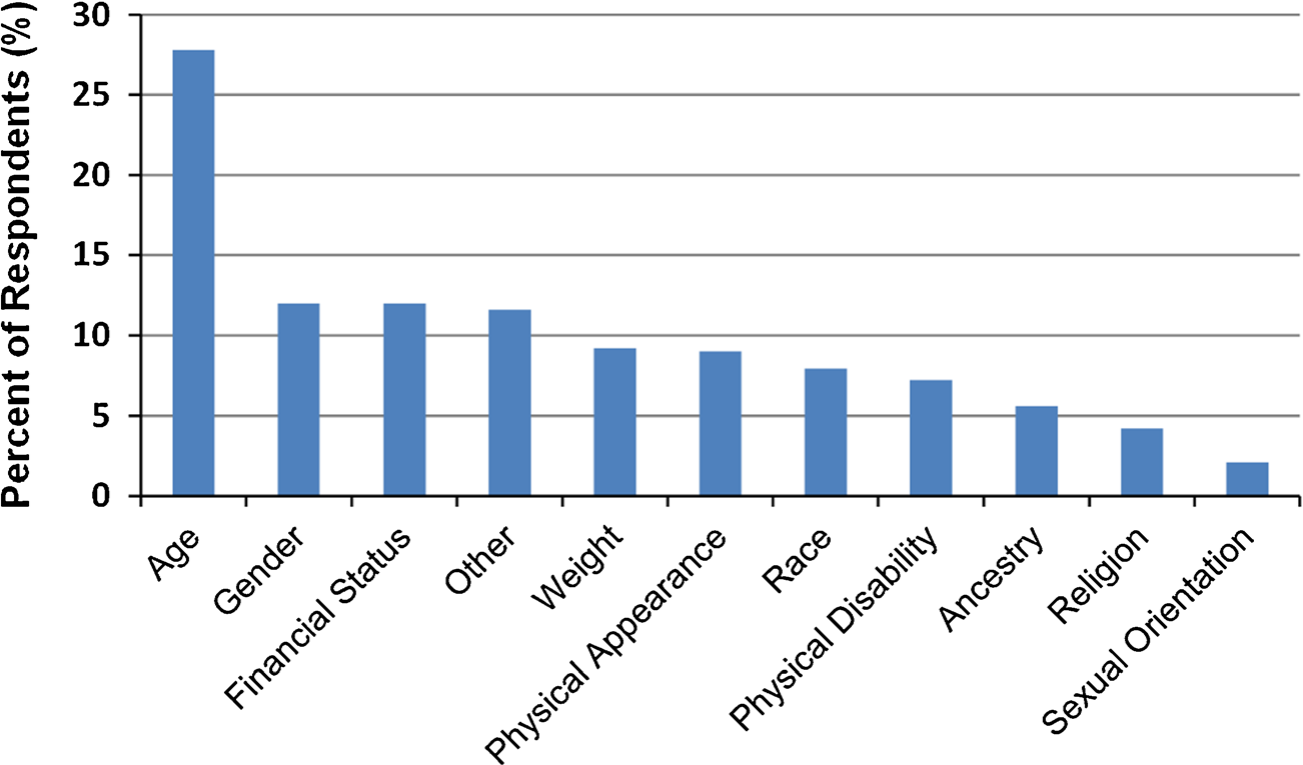
Reasons for discrimination reported by those experiencing healthcare discrimination.* *Legend: Given the high degree of overlap between healthcare discrimination and affirmative responses to other items on the Everyday Discrimination Scale, reasons for discrimination should be interpreted as general reasons for discrimination, rather than reasons specific to the healthcare setting.

Figure 1 presents the reasons participants who reported healthcare discrimination selected to explain why they experienced discrimination. The most common reasons given by those who experienced healthcare discrimination were age (28 %), gender (12 %), and financial status (12 %) and the top three reasons were similar whether they experienced discrimination infrequently (age 49 %, financial status 25 %, gender 22 %) or frequently (age 53 %, financial status 35 %, or gender 22 %). Age was the most common reason for all age groups, both genders, those with ADL difficulty, and most race/ethnic groups, with at least one-quarter of all participants marking it as a significant reason. The exception was African American participants, who reported race (45.9 %) as the most common reason. Even among this group, however, over one-quarter (28 %) reported age as a reason.

### Healthcare Discrimination and Disability

**Figure 2.**
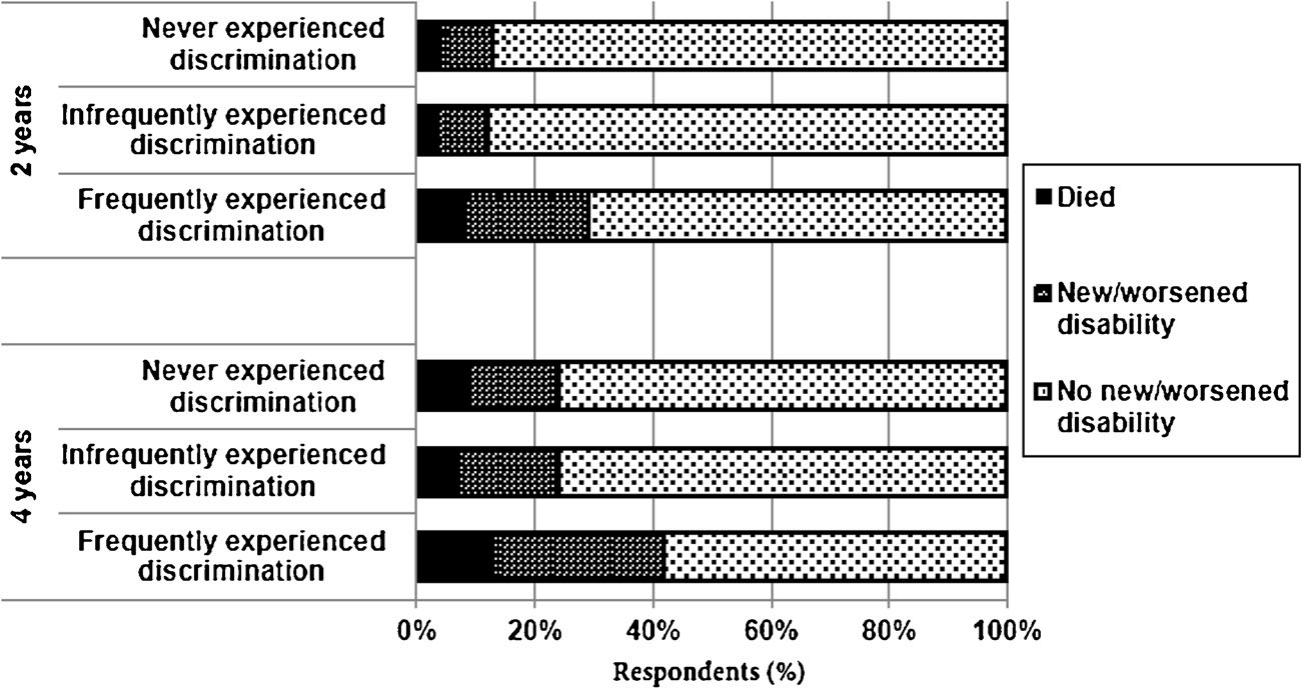
Change in functional status of participants 2 and 4 years after reporting healthcare discrimination.

**Figure 3.**
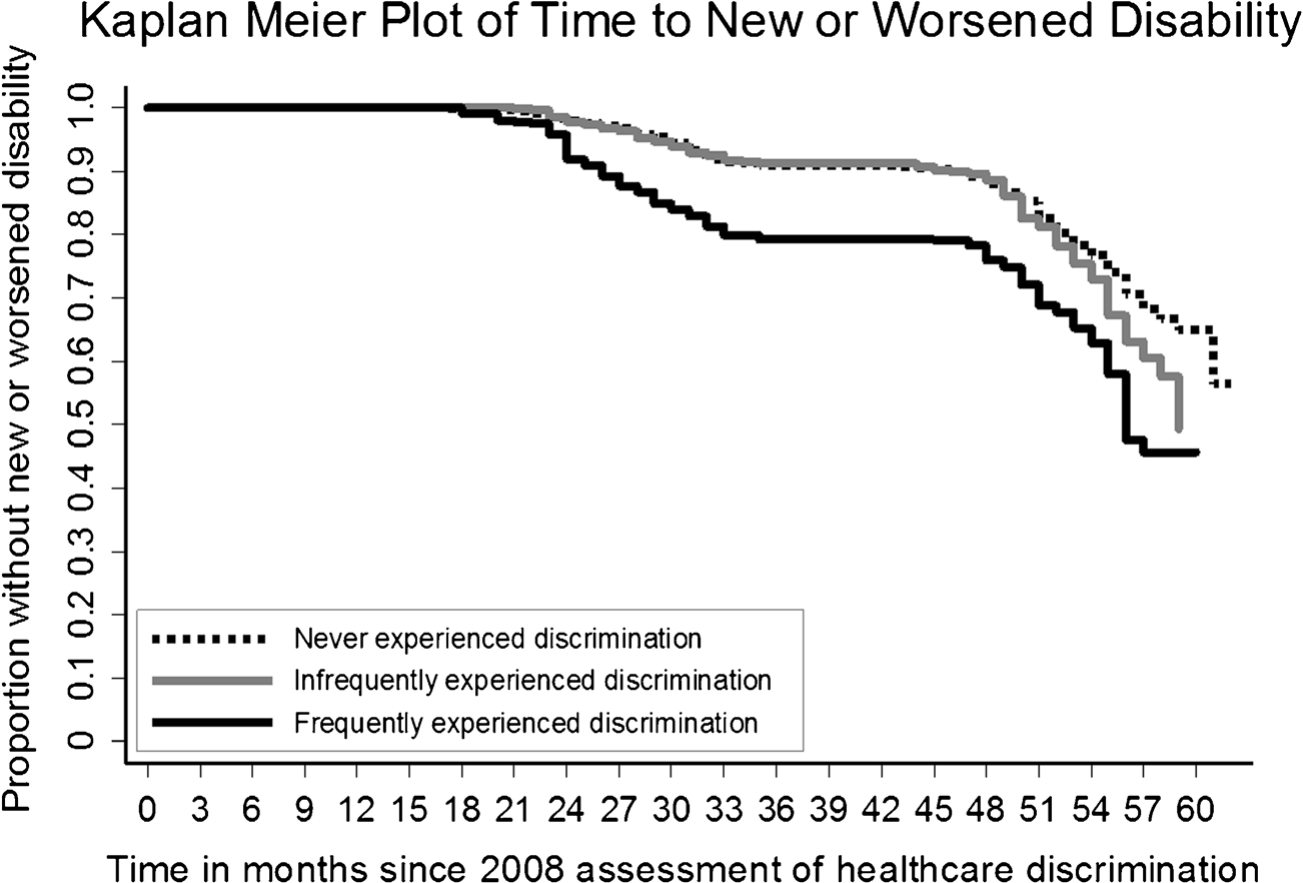
Legend: Follow-up interviews were conducted every 2 years, in 2010 and 2012, although not precisely at 2-year intervals. The two plateaus in the plot correspond to the time between participant interviews.

Four years after the assessment for healthcare discrimination, 28.5 % (95 % CI, 22.2–35.9) of participants that experienced frequent discrimination reported a new or worsened disability, compared to 14.7 % (95 % CI, 13.6–16.0) of those who never experienced discrimination and 16.8 % (95 % CI 13.8–20.3) of those infrequently experiencing discrimination (*p* < 0.001). Participants who never or infrequently experienced discrimination were more likely to maintain their functional status at 2 and 4 years (Fig. 2). At 2 years, those who frequently experienced discrimination were significantly more likely to have new or worsened disability (20.7 %), compared to those who infrequently experienced discrimination (8.7 %) and those who did not experience it (9 %) (*p* < 0.001). Fig. 3 shows a Kaplan Meier plot, indicating that differences in functional decline between those who experienced discrimination frequently and other groups that appeared at the time of the first follow-up interview in 2010.

After adjustment for sociodemographic factors, comorbidities, and healthcare utilization, participants who frequently experienced discrimination were 63 % more likely to experience new or worsened disability within 4 years compared to those who experienced no healthcare discrimination (aHR 1.63, 95 % CI, 1.16–2.27) (Table 2).

**Table 2.** Healthcare Discrimination and its Association with Disability by 2012

Reported healthcare discrimination in 2008	Risk of new or worsened disability by 2012*		
	Unadjusted	Adjusted for age groups	Adjusted for sociodemographic factors, comorbidities, and healthcare system utilization**
	Hazard Ratio (95 % CI)	Hazard Ratio (95 % CI)	Hazard Ratio (95 % CI)
Never experienced discrimination	–	–	–
Infrequently experienced discrimination	1.12 (0.90–1.40) [*p* = 0.32]	1.22 (0.98–1.52) [*p* = 0.08]	1.09 (0.85–1.40) [*p* = 0.48]
Frequently experienced discrimination	2.07 (1.51–2.85) [*p* < 0.001]	2.28 (1.66–3.15) [*p* < 0.001]	1.63 (1.16–2.27) [*p* = 0.005]

Reported values incorporate survey weights to account for the complex survey design

*Defined participants who died in follow-up without ADL evaluations as ‘No New/Worsen Disability’

**Demographic factors include: age groups, gender, race/ethnicity, education, and net worth. Comorbidities include: high blood pressure, diabetes, cancer, lung disease, heart disease, stroke, and depression. Healthcare system utilization includes: hospitalizations, nursing home stays, outpatient surgery visits, clinic or emergency department visits, and use of home health services in the past two years (2006–2008)

### Everyday Discrimination and Disability

Those who reported frequent healthcare discrimination were very likely to respond positively to other items on the EDS (range 53–91 %) (See Table 3, online appendix). However, participants who reported frequent discrimination in response to other items tended to not report frequent discrimination in healthcare settings (range 38–54 %) (Table 3, online appendix). In our sub-analysis, we found no association between frequent everyday discrimination outside of healthcare settings and disability. Specifically, we first compared the development of disability over 4 years between the 2,418 participants who reported any frequent discrimination in response to any measure on the EDS and those 2,297 participants who reported no discrimination in any aspect of their life, and found no association (aHR 1.06, 95 % CI 0.89–1.27, *p* = 0.51). Secondly, we compared the development of disability between the 2,374 participants who reported frequent non-healthcare discrimination using any other measure on the EDS other than the item about healthcare discrimination to the 2,328 participants who did not report non-healthcare discrimination, and found no association (aHR 1.08, 95 % CI, 0.90–1.29, *p* = 0.42).

## DISCUSSION

Discrimination in healthcare settings is common, and is experienced by one out of five older adults over the age of 50 years. We found an association between those who experienced healthcare discrimination more than once per year, as they were more likely to develop new or worsened disability within 4 years compared to those who reported no such experiences. This finding persisted after accounting for confounders, including sociodemographic factors, comorbid conditions, and healthcare system utilization. In addition, our study found that healthcare discrimination is distinct from discrimination outside of healthcare settings, in that healthcare discrimination is associated with the development of disability and discrimination outside of healthcare settings is not. To our knowledge, this is the first study to find the association between disability and self-reported healthcare discrimination using a nationally representative sample of older adults.

What is as yet unknown is the precise relationship between reported discrimination in healthcare settings and the development of disability. Is this relationship causal? The longitudinal nature of our study, with the presence of the risk factor measured prior to the onset or worsening of disability, lends strength to the causal inference. However, this association may well exist because of an association of healthcare discrimination with other factors that are not captured in our multivariable model that lead to disability. Furthermore, our measure of healthcare discrimination included worse care from doctors or hospitals. It is not clear, therefore, if reported discrimination was due more to experiences with doctors, and/or discrimination by hospitals (including other members of the healthcare team), and/or the healthcare system as a whole.

If we hypothesize for the moment that the relationship is causal, also unknown are the precise mechanisms by which discrimination experienced by older adults in healthcare settings lead to worsened disability. In healthcare settings, discrimination has been linked to less psychosocial communication in doctors’ visits, a decrease in the ease of communication as perceived by the patient, and decreased informativeness.^29^ Experiencing discrimination is associated with decreased cancer screening,^18^ fewer provider visits,^30^ decrease in some preventative health services,^31^ a delay in filling prescriptions, and a delay in testing and treatment.^32^ In one recent study, fictitious obese or disabled patients were denied access to care, with some facilities stating they could not accommodate or transfer the patient, or that the building was inaccessible.^33^ Future observational research should attempt to test the robustness of this association, explore the sources of discrimination (i.e., physicians vs. non-physician staff vs. hospital or health systems), and examine potential mechanisms by which discrimination in healthcare settings leads to disability.

Health care systems are complex, as exemplified by the IHI triple aim of: 1) improving the patient experience of care; 2) improving the health of populations; 3) reducing the per capita cost of health care.^1^ This complex set of aims necessarily produces a complex relationship between health systems, healthcare providers, and patients. Factors that lead to reduced costs of care, such as high co-pays for brand name medications, may be at odds with factors that lead to an improved patient experience, such as access to preferred medications. Factors that improve the health of populations, such as a physician reporting an unsafe elderly driver, may create friction with the patient that leads to perceived differential treatment on the basis of age. Our data suggest that—at best—our providers and health systems are failing to adequately explain the rationale for the choices they make as they strive to simultaneously improve patient care, health, and efficiency. At worst, there may be actual discrimination that leads to worse outcomes, such as disability, for patients over time.

Additional limitations are noted. Healthcare discrimination is certainly multidimensional and complex, and we were limited to a single measure. There is some debate as to whether patients’ reports of discrimination are adequate measures of actual discrimination on the part of the healthcare system. However, patients’ experiences with care are the gold standard when assessing patient satisfaction in clinical settings.^34^ Nevertheless, it may be that the perception of discrimination itself that contributes to poor outcomes.^10^ In addition, one might argue that older adults who use more healthcare resources are at greatest risk of becoming disabled. These individuals who are sicker and use the healthcare system frequently may report poorer service by doctors or hospitals simply because they have more opportunities to perceive discrimination. For this reason, we adjusted for frequency of contact with the healthcare system.

## CONCLUSION

Reducing experiences of discrimination and the perception of discrimination should be a priority in its own right. Additionally, research to sort out the reasons, sources, and precise nature of the relationship between healthcare discrimination and the development of disability should be a priority. Hopefully, we will find that reducing experiences of discrimination improves the function and wellbeing of older adults, sparing them, their family, caregivers, and society financial, physical and emotional distress. Providers and healthcare workers can aim to treat each patient with respect, dignity and fairness, regardless of age, race, or socioeconomic status, and this in turn may improve the function of our aging society.

## Electronic supplementary material

Below is the link to the electronic supplementary material.

eTable 3 (DOC 44 kb)
