# Supplementary material for: Discrimination in Healthcare Settings is Associated with Disability in Older Adults: Health and Retirement Study, 2008–2012
Source: J Gen Intern Med. 2015 Mar 13;30(10):1413–20. doi: 10.1007/s11606-015-3233-6 (PMC4579241; doi:10.1007/s11606-015-3233-6)
Supplement: Supplementary file 1 — (DOC 44 kb) [file 11606_2015_3233_MOESM1_ESM.doc]

**eTable 3. Frequency and Percentage of Participants Who Reported Any Frequent Discrimination on the Everyday Discrimination Scale**

|  | **Be Treated with Less Respect**  **(n=2047)** | **Receive Poorer Service than Others**  **(n=1541)** | **People Act as if You not Smart**  **(n=1846)** | **People Act as if Afraid of You**  **(n=895)** | **You Are Threatened or Harassed**  **(n=770)** | **Worse Treatment From Doctors or Hospitals**  **(n=846)** |
| --- | --- | --- | --- | --- | --- | --- |
| **Be Treated with Less Respect** | *2047 (100.0%)* | 1429 (94.1%) | 1590 (87.5%) | 783 (86.2%) | 702 (91.3%) | 764 (91.3%) |
| **Receive Poorer Service than Others** | 1429 (71.8%) | *1541 (100.0%)* | 1292 (71.3%) | 661 (72.1%) | 592 (78.3%) | 707 (86.1%) |
| **People Act as if You not Smart** | 1590 (79.4%) | 1292 (84.7%) | *1846 (100.0%)* | 728 (81.2%) | 662 (86.8%) | 722 (86.5%) |
| **People Act as if Afraid of You** | 783 (42.1%) | 661 (46.1%) | 728 (43.7%) | *895 (100.0%)* | 474 (65.5%) | 425 (52.8%) |
| **You Are Threatened or Harassed** | 702 (36.9%) | 592 (41.5%) | 662 (38.7%) | 474 (54.2%) | *770 (100.0%)* | 422 (52.8%) |
| **Worse Treatment From Doctors or Hospitals** | 764 (38.0%) | 707 (46.8%) | 722 (39.7%) | 425 (45.1%) | 422 (54.1%) | *846 (100.0%)* |

Reported values incorporate survey weights to account for the complex survey design.
